# Supplementary material for: Mapping combinatorial drug effects to DNA damage response kinase inhibitors
Source: Nat Commun. 2023 Dec 14;14:8310. doi: 10.1038/s41467-023-44108-y (PMC10721915; doi:10.1038/s41467-023-44108-y)

Supplementary materials of Zhang et al. Mapping the molecular dependencies of combination therapeutic response to DNA damage response kinase inhibitors

Supplementary Data Legends

Supplementary Data 1. Target gene and mode-of-action of all anti-cancer drugs tested in this study.

Supplementary Data 2. Top ten directly targeted genes (among the 272 genes directly targeted by all drugs in this study) that achieved the highest efficacy (AoC score) across all cell lines in combination with the inhibition of drug targets ATM, ATR, or DNA-PK (PRKDC) using the best model predicting across cell lines. **: genes that occur in the top ten in combination with all three drug targets. *: genes that occur in the top ten in combination with two out of three drug targets.

Supplementary Data 3. Top ten directly targeted genes (among the 272 genes directly targeted by all drugs in this study) that achieved the highest synergy (Bliss score) across all cell lines in combination with the inhibition of drug targets ATM, ATR, or DNA-PK (PRKDC). **: genes that occur in the top ten in combination with all three drug targets. *: genes that occur in the top ten in combination with two out of three drug targets.

Supplementary Data 4. Statistical analysis of tissue specificity on monotherapies. Supplementary Data 4-1. The results of all monotherapies from non-parametric omnibus Kruskal-Wallis tests. Supplementary Data 4-2 to 4-5 . The results generated by the post-hoc Dunn's test (Supplementary Data 4-2), Mann-Whitney test (Supplementary Data 4-3), Conover-Iman test (Supplementary Data 4-4) and bootstrapping (Supplementary Data 4-5) on the significant ($p < 0.0001$, two-sided) samples from the Kruskal-Wallis test.

Supplementary Data 5. Statistical analysis of tissue specificity on combination therapies. Supplementary Data 5-1. The results of the efficacy of all combination therapy from non-parametric omnibus Kruskal-Wallis tests. Supplementary Data 5-2. The results of the synergy of all combination therapy from non-parametric omnibus Kruskal-Wallis tests. Supplementary Data 5-3 to Supplementary Data 5-6. The post-hoc Dunn's test (Supplementary Data 5-3), Mann-Whitney test (Supplementary Data 5-4), Conover-Iman test (Supplementary Data 5-5) and bootstrapping (Supplementary Data 5-6) on the efficacy of the significant ($p < 0.001$) samples from the Kruskal-Wallis test. Supplementary Data 5-7 to Supplementary Data 5-10. The post-hoc Dunn's test (Supplementary Data 5-7), Mann-Whitney test (S5-8), Conover-Iman test (Supplementary Data 5-9) and bootstrapping (Supplementary Data 5-10) on the synergy of the significant ($p < 0.001$, two-sided) samples from the Kruskal-Wallis test.

Supplementary Figures

Supplementary Figure 1. Overview of all monotherapies used in this study. (**a**) the significance (-log$_{10}$(p)) from the Kruscal-Wallis variance test across all cancer types for each monotherapy. A dashed line marks the significance threshold (p=0.01, two-sided). (**b**) The total count of experiments of monotherapy. (**c**) boxplot shows the efficacy of all anti-cancer drugs used in this study. The color of the boxplot indicated the mode-of-actions. Drugs were ordered by average efficacy in all experiments in descending order.

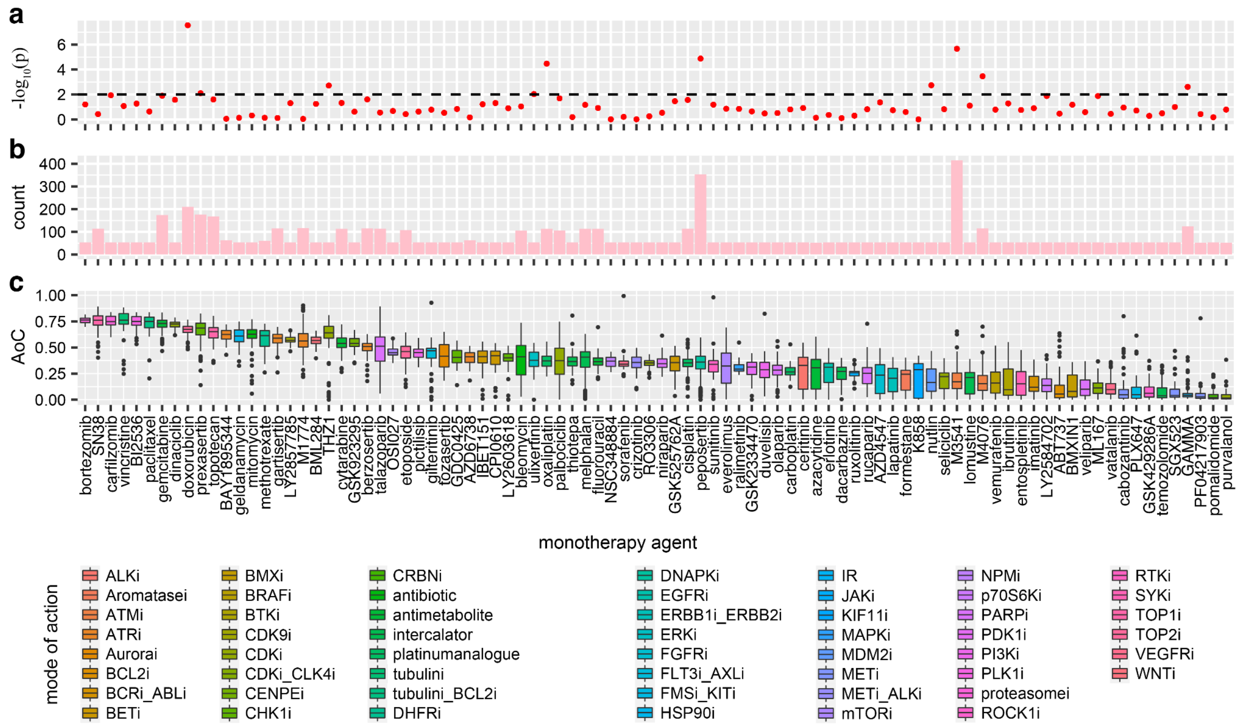

Supplementary Figure 2. Heatmap shows the results from post-hoc analysis on the significantly variant monotherapy and combination treatments from the Kruscal-Wallis test, and the right lane shows the distribution of response score (AoC or Bliss) in different cancer types. (**a**) shows post-hoc analysis results of monotherapy doxorubicin, M3541, peposertib, and oxaliplatin, respectively**., and** (**b**) shows post-hoc analysis results of combination therapy peposertib-gamma-ionizing-radiation (AoC and Bliss score) and M4076-berzosertib (Bliss score). Boxplots show the 25, 50 and 75 percentiles with with whiskers extending to 1.5 times the interquartile range; for each cancer types the total numbers of cell lines are: bladder=4; brain=3; breast=6; colon=8; hematological=10; liver=2; lung=5; melanoma=3; ovary=5; pancreas=4; prostate=2; sarcoma=10. All statistically significant values from the variance test are two-sided.

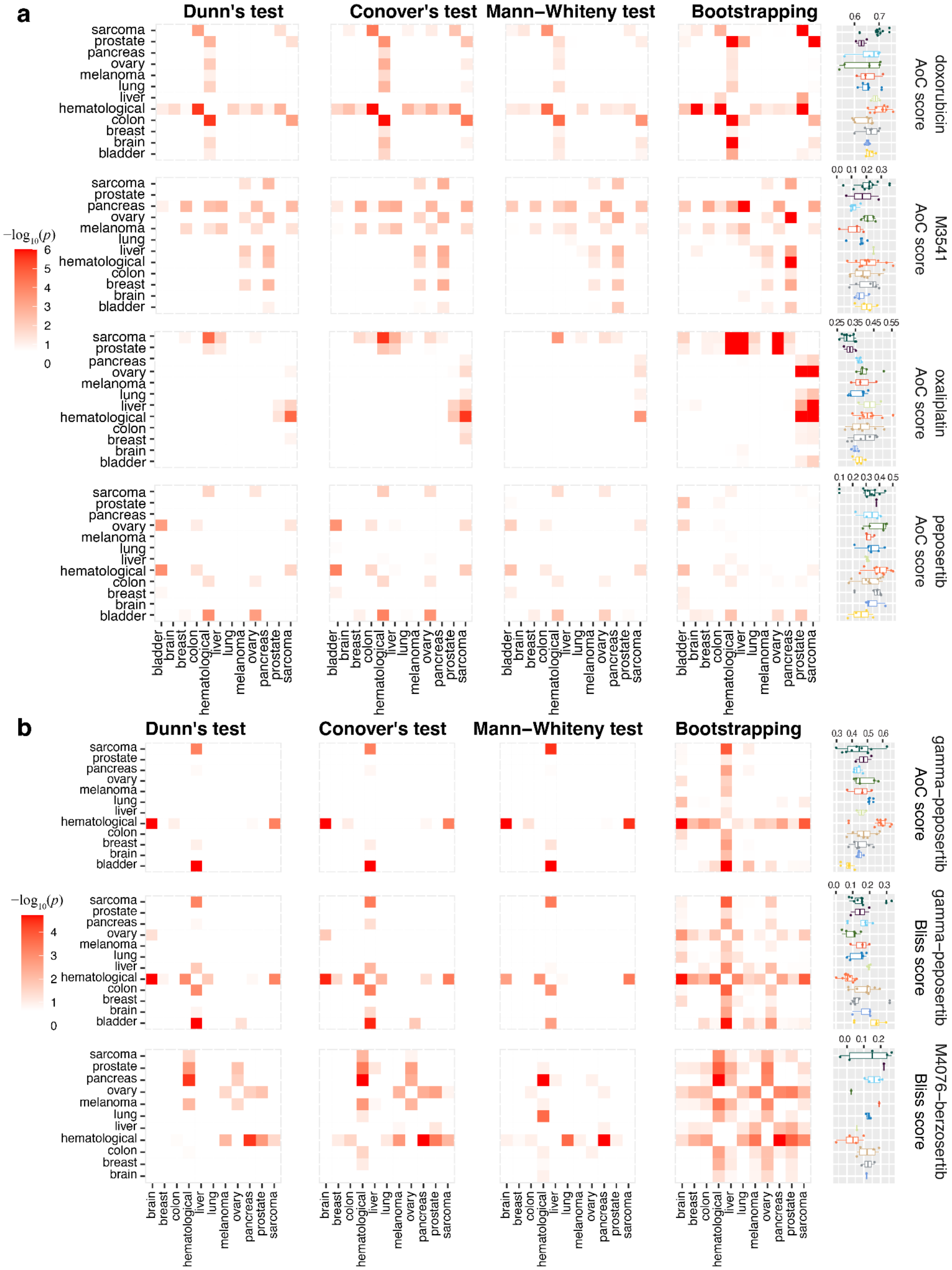

Supplementary Figure 3. Hierarchy clustering of monotherapy from responses (efficacy) on different cell lines.

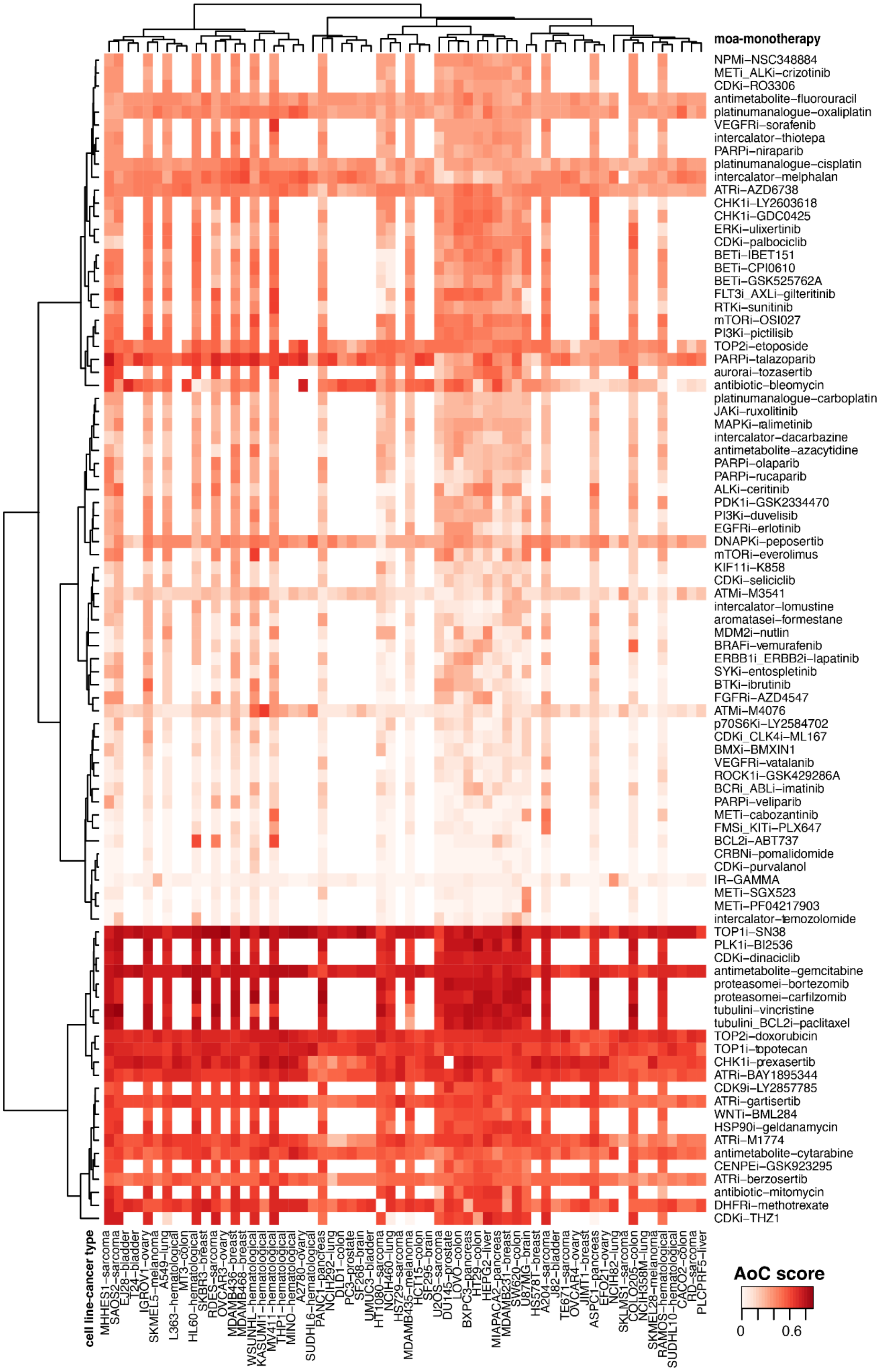

Supplementary Figure 4. Hierarchy clustering of combinations from responses (efficacy) on different cell lines.

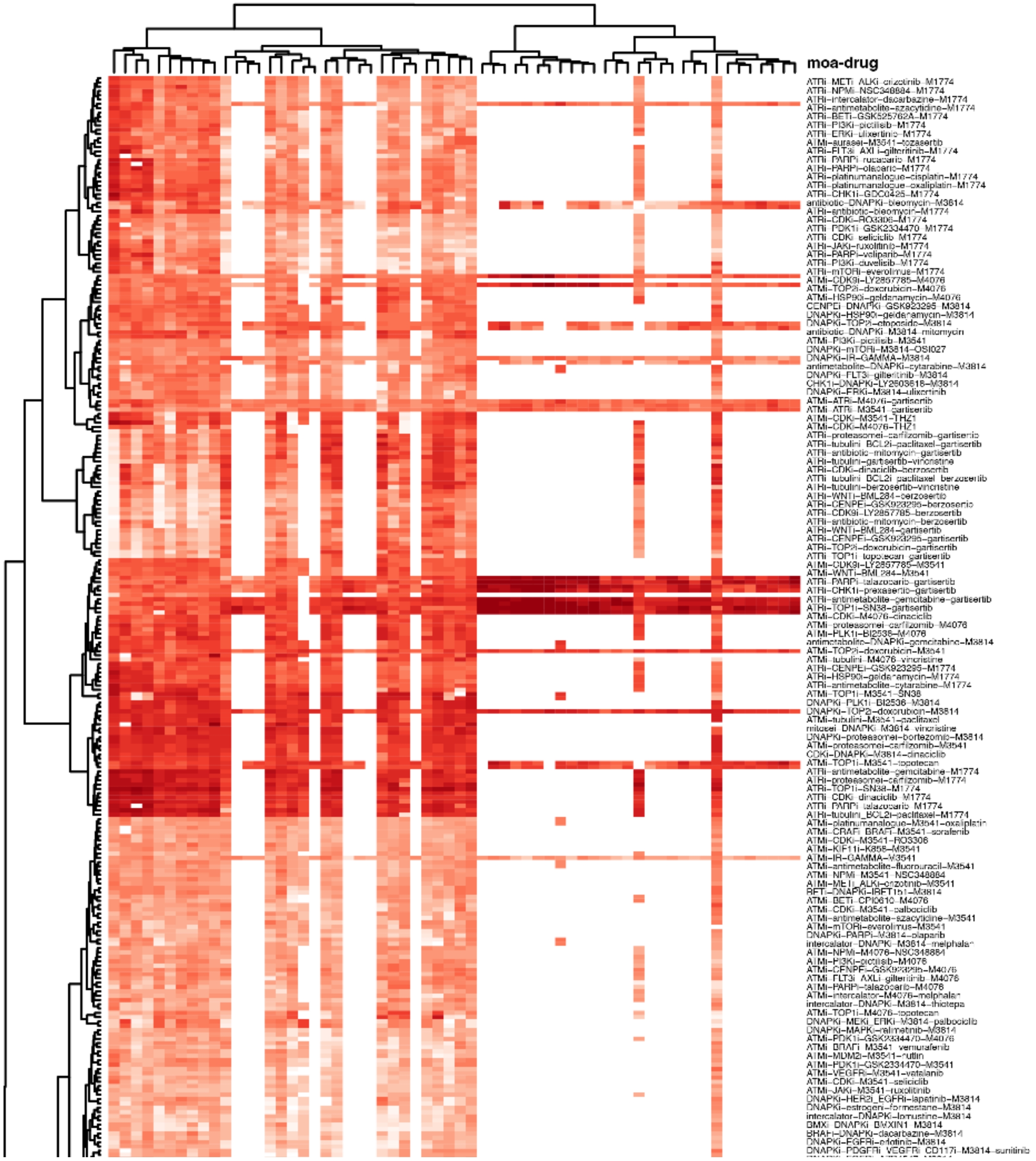

Supplementary Figure 5. Hierarchy clustering of combinations from responses (efficacy) on different cell lines.

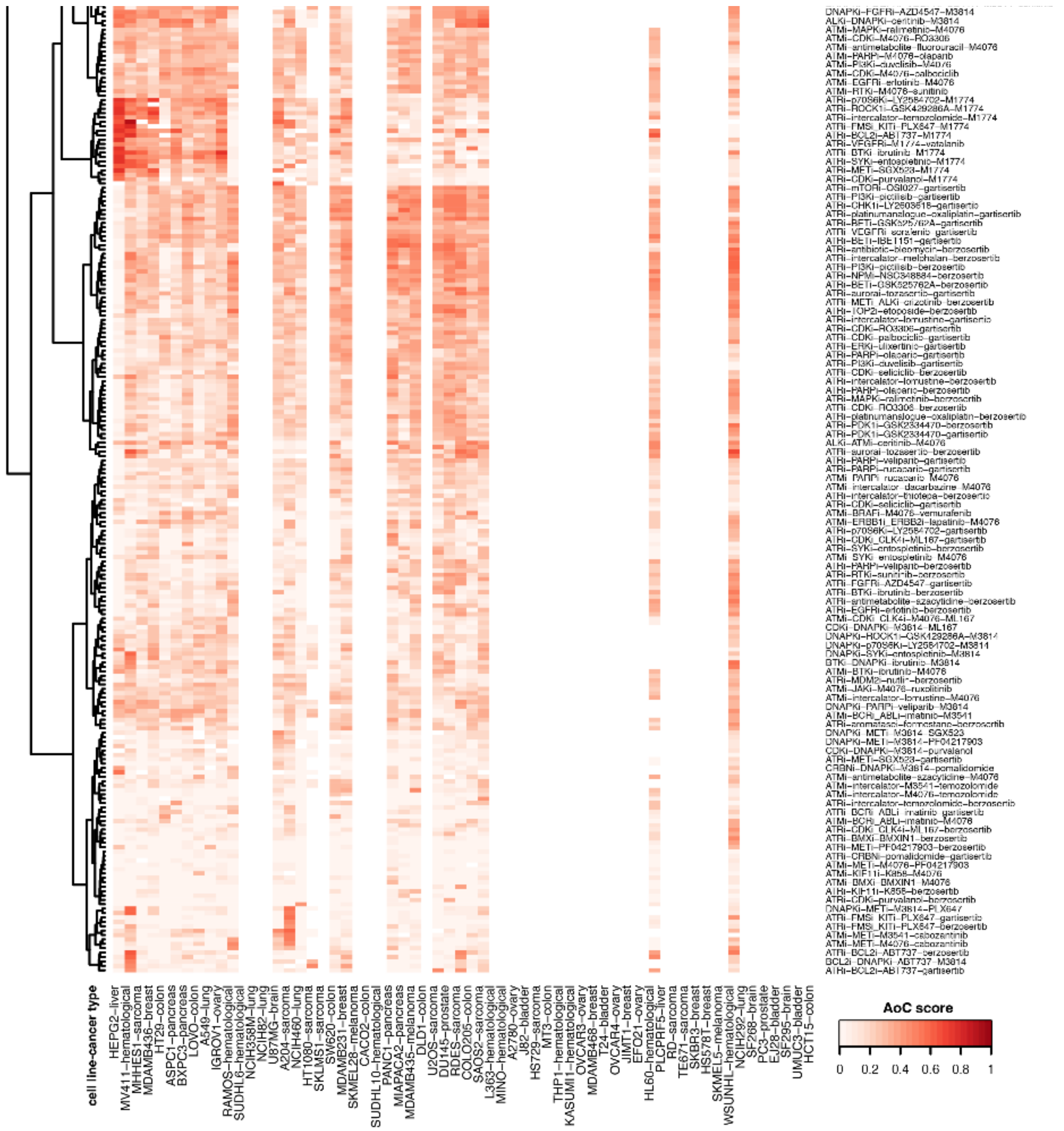

Supplementary Figure 6. Hierarchy clustering of combinations from responses (synergy) on different cell lines.

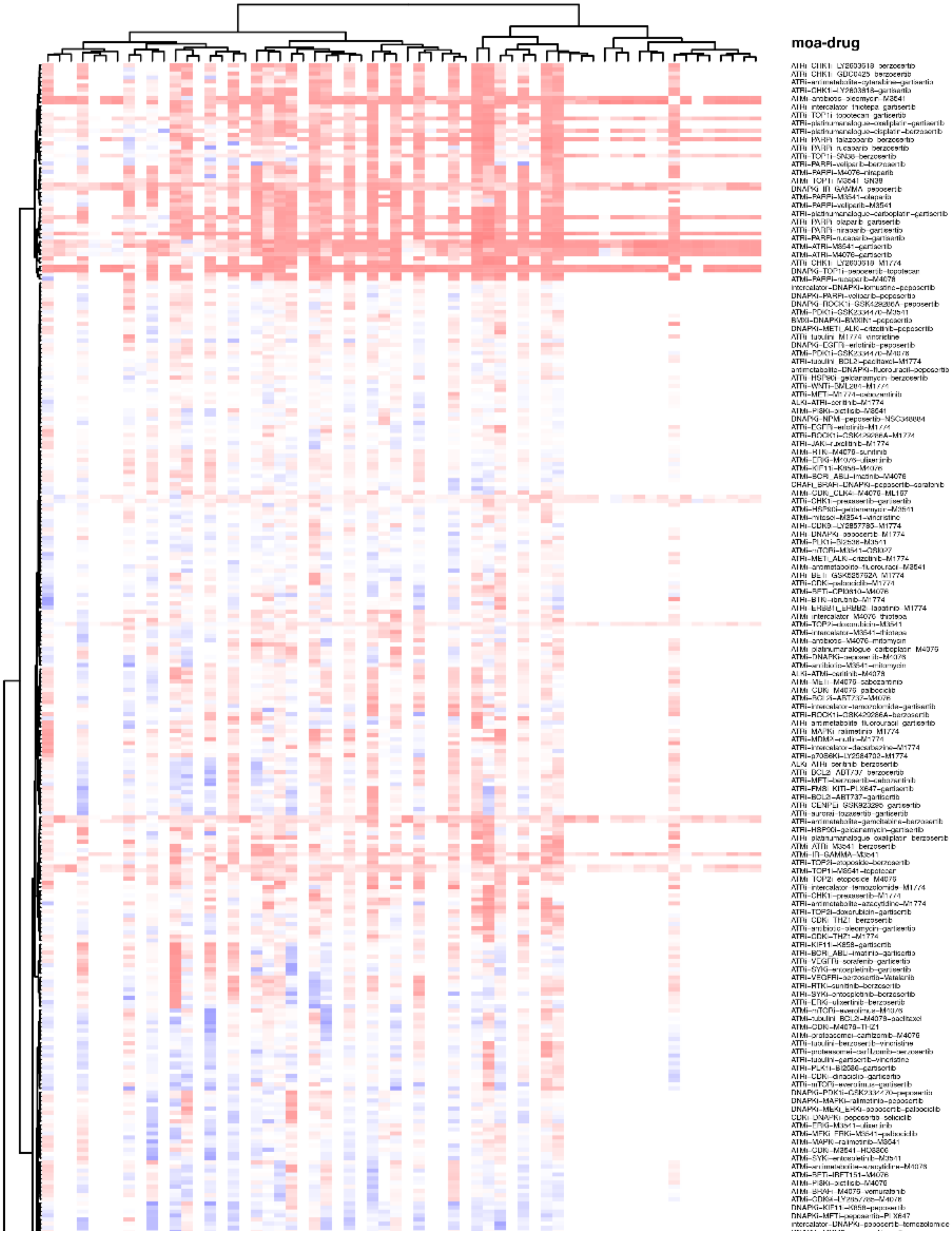

Supplementary Figure 7. Hierarchy clustering of combinations from responses (synergy) on different cell lines.

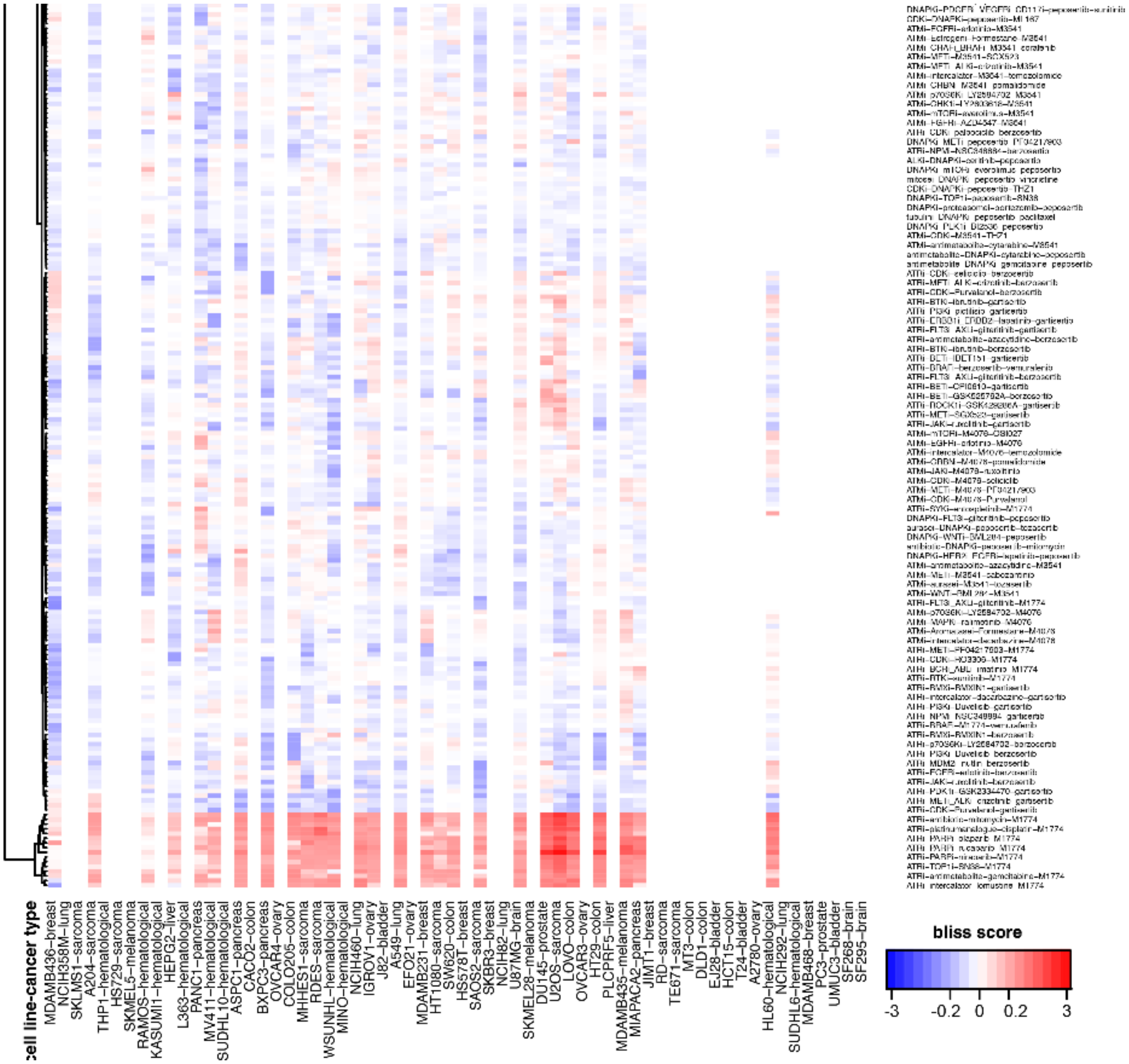

Supplementary Figure 8. Demonstration of the dose-response matrices of peposertib-gamma-ionizing-radiation combination treatment. The responses (growth inhibition rate, GI) in cell lines at different doses of peposertib (mol) and gamma ionizing-radiation (Gy) were shown by heatmaps.

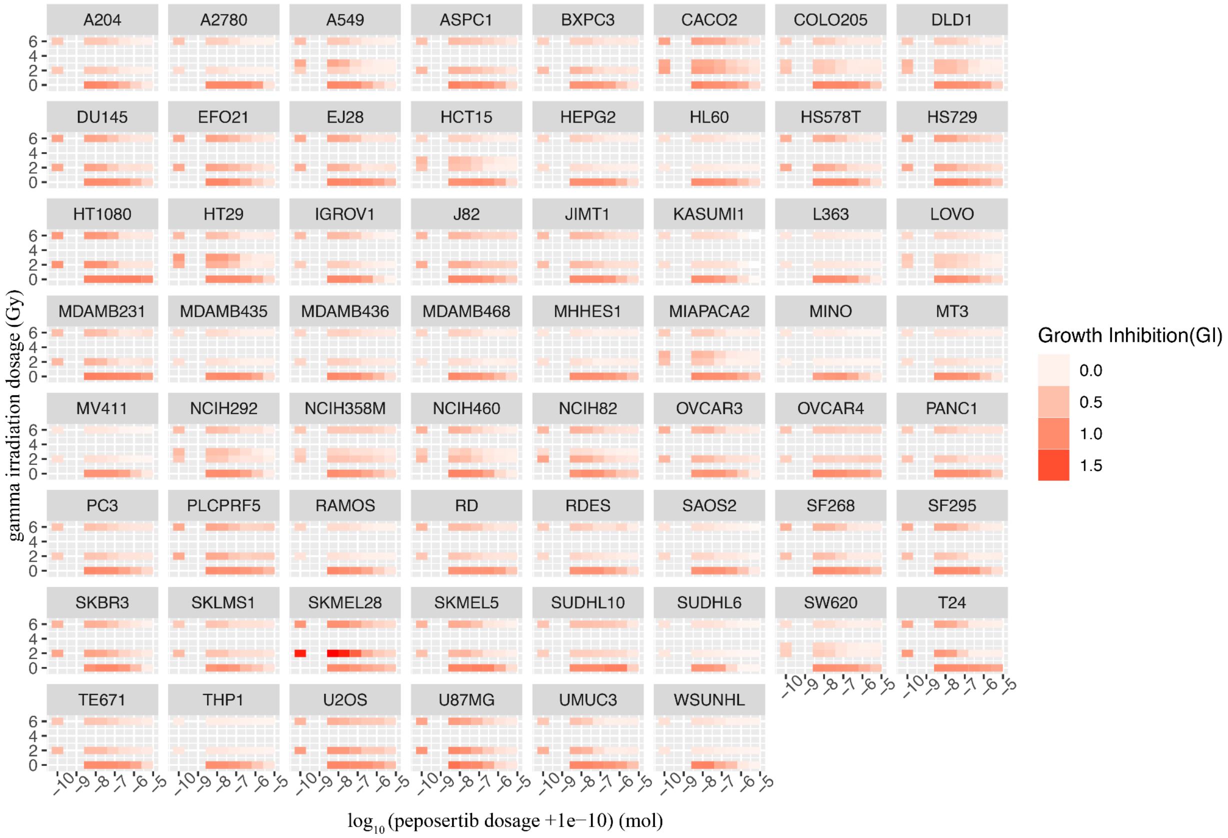

Supplementary Figure 9. Demonstration of the dose-response matrices of M4076-berzosertib combination treatment. The responses (growth inhibition rate, GI) in cell lines at different doses of M4076 (mol) and berzosertib (mol) were shown by heatmaps.

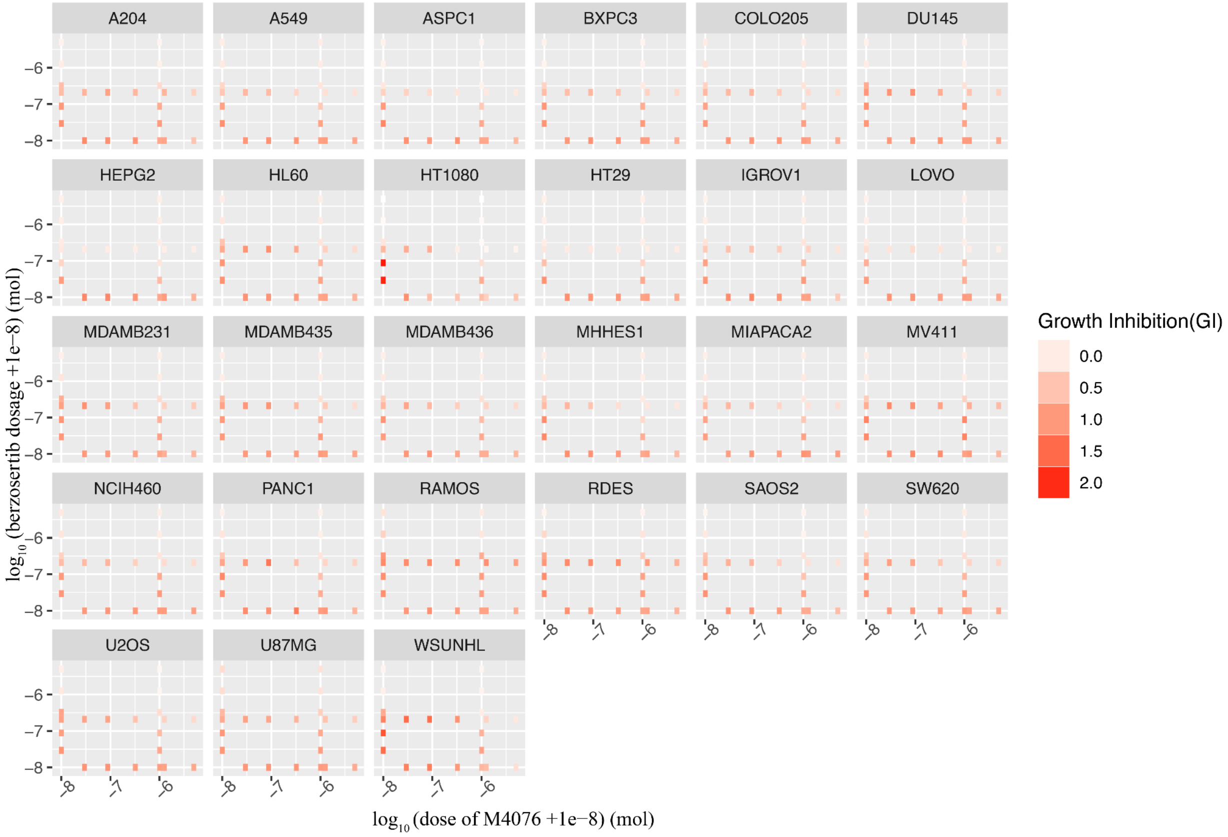

Supplement: Supplementary file 1 — Supplementary information [file 41467_2023_44108_MOESM1_ESM.pdf]
