# Supplementary material for: Mapping combinatorial drug effects to DNA damage response kinase inhibitors
Source: Nat Commun. 2023 Dec 14;14:8310. doi: 10.1038/s41467-023-44108-y (PMC10721915; doi:10.1038/s41467-023-44108-y)
Supplement: Supplementary file 3 — Description of Additional Supplementary Files [file 41467_2023_44108_MOESM3_ESM.pdf]

Description of Additional Supplementary Files

Supplementary Data 1. Target gene and mode-of-action of all anti-cancer drugs tested in this study.

Supplementary Data 2. Top ten directly targeted genes (among the 272 genes directly targeted by all drugs in this study) that achieved the highest efficacy (AoC score) across all cell lines in combination with the inhibition of drug targets ATM, ATR, or DNA-PK (PRKDC) using the best model predicting across cell lines. **: genes that occur in the top ten in combination with all three drug targets. *: genes that occur in the top ten in combination with two out of three drug targets.

Supplementary Data 3. **Top ten directly targeted genes (among the 272 genes directly targeted by all drugs in this study) that achieved the highest synergy (Bliss score) across all cell lines in combination with the inhibition of drug targets ATM, ATR, or DNA-PK (PRKDC).** **: genes that occur in the top ten in combination with all three drug targets. *: genes that occur in the top ten in combination with two out of three drug targets.

Supplementary Data 4. Statistical analysis of tissue specificity on monotherapies. Table S4-1. The results of all monotherapies from non-parametric omnibus Kruskal-Wallis tests. Table S4-2 to S4-5. The results generated by the post-hoc Dunn's test (S4-2), Mann-Whitney test (S4-3), Conover-Iman test (S4-4) and bootstrapping (S4-5) on the significant ($p < 0.0001$, two-sided) samples from the Kruskal-Wallis test.

Supplementary Data 5. Statistical analysis of tissue specificity on combination therapies. Table S5-1. The results of the efficacy of all combination therapy from non-parametric omnibus Kruskal-Wallis tests. Table S5-2. The results of the synergy of all combination therapy from non-parametric omnibus Kruskal-Wallis tests. Table S5-3 to S5-6. The post-hoc Dunn's test (S5-3), Mann-Whitney test (S5-4), Conover-Iman test(S5-5) and bootstrapping (S5-6) on the efficacy of the significant ($p < 0.001$) samples from the Kruskal-Wallis test.

Table S5-7 to S5-10. The post-hoc Dunn's test (S5-7), Mann-Whitney test (S5-8), Conover-Iman test (S5-9) and bootstrapping (S5-10) on the synergy of the significant ($p < 0.001$, two-sided) samples from the Kruskal-Wallis test.

Supplementary Data 6. Versions of all softwares in this study.
