# Supplementary material for: Mapping combinatorial drug effects to DNA damage response kinase inhibitors
Source: Nat Commun. 2023 Dec 14;14:8310. doi: 10.1038/s41467-023-44108-y (PMC10721915; doi:10.1038/s41467-023-44108-y)
Supplement: Supplementary file 9 — Reporting Summary [file 41467_2023_44108_MOESM9_ESM.pdf]

nature portfolio

Corresponding author(s): Yuanfang Guan

Last updated by author(s): Oct 19, 2023

Reporting Summary

Nature Portfolio wishes to improve the reproducibility of the work that we publish. This form provides structure for consistency and transparency in reporting. For further information on Nature Portfolio policies, see our Editorial Policies and the Editorial Policy Checklist.

Statistics

For all statistical analyses, confirm that the following items are present in the figure legend, table legend, main text, or Methods section.

n/a	Confirmed	
☐	☒	The exact sample size (*n*) for each experimental group/condition, given as a discrete number and unit of measurement
☐	☒	A statement on whether measurements were taken from distinct samples or whether the same sample was measured repeatedly
☐	☒	The statistical test(s) used AND whether they are one- or two-sided<br>*Only common tests should be described solely by name; describe more complex techniques in the Methods section.*
☐	☒	A description of all covariates tested
☐	☒	A description of any assumptions or corrections, such as tests of normality and adjustment for multiple comparisons
☐	☒	A full description of the statistical parameters including central tendency (e.g. means) or other basic estimates (e.g. regression coefficient) AND variation (e.g. standard deviation) or associated estimates of uncertainty (e.g. confidence intervals)
☐	☒	For null hypothesis testing, the test statistic (e.g. *F*, *t*, *r*) with confidence intervals, effect sizes, degrees of freedom and *P* value noted<br>*Give P values as exact values whenever suitable.*
☒	☐	For Bayesian analysis, information on the choice of priors and Markov chain Monte Carlo settings
☒	☐	For hierarchical and complex designs, identification of the appropriate level for tests and full reporting of outcomes
☐	☒	Estimates of effect sizes (e.g. Cohen's *d*, Pearson's *r*), indicating how they were calculated

Our web collection on statistics for biologists contains articles on many of the points above.

Software and code

Policy information about availability of computer code

Data collection

All dose-response experiments were conducted at Oncolead GmbH & Co. KG (Karlsfeld, Germany). Cell lines were purchased directly from the ATCC, NCI, CLS and DSMZ cell line collections. The cell lines were grown in the media recommended by the suppliers in the presence of 100 U/ml penicillinG and 100 µg/ml streptomycin supplied with 10% FCS.

Cells were grown in a 5% CO2 atmosphere. Cell growth and treatment were performed in 96-well microtiter plates CELLSTAR® (Greiner Bio-One, Germany). Cells harvested from exponential phase cultures by trypsinization or by splitting (in the case of suspension growing cells) were plated in 90 µl of media at optimal seeding densities. The optimal seeding density for each cell line was determined to ensure exponential growth for the duration of the experiment. All cells growing without anticancer agents were sub-confluent by the end of the treatment, as determined by visual inspection.

Cells were allowed to stay for another 48 hours prior to compound treatment. The treatment was performed for 120 hours and stopped by the addition of trichloroacetic acid followed by using a total protein staining protocol (Sulforhodamine B (SRB) staining) 60. The bound SRB was solubilized with 100 µl of 10 mM Tris base. Optical density was measured at 492, 520, and 560 nm. Compound dilutions were performed in DMSO and diluted 1:100 in the RPMI medium. Combined treatment has been performed simultaneously. 90 µl of cells were treated by mixing with 10 µl of the compound-containing media (resulting in a final DMSO concentration of 0.1%). In the case of combination, both agents were mixed together in DMSO at equal volumes so that the final concentration of DMSO was 0.2%. In addition, all experiments contained a few plates with cells that were analyzed immediately after the 48 hours recovery period. These plates contained information about the cell number, Tz, at time zero, i.e., before treatment, and served to calculate the cytotoxicity.

The calculation nomenclature used was introduced by DTP of the NCI 61. The first step in data processing was calculating an average background value for each plate, derived from plates and wells containing mediums without cells. The average background optical density was then subtracted from the appropriate control values (containing cells without the addition of a drug), from values representing the cells treated with an anticancer agent, and from values of wells containing cells at time zero. Thus, the following values were obtained for each experiment: control cell growth, C; cells in the presence of an anticancer agent Ti and cells prior to compound treatment at time zero, Tz (or

	T0, in some publications).<br>The selection of the concentration range for all agents was based on previous experiments using a panel of 62 cell lines. A 4-fold dilution and 5 data points were sufficient to cover the complete activity range for most of the agents (Fig. S6 and 7).
Data analysis	Dose-response evaluation measures<br>The non-linear curve fitting calculations were performed using algorithms and visualization tools using four-parameter log-logistic regression 62,63.<br>To obtain an estimate of treatment efficacy that encompasses both potency and maximum effect, the relative area over the curve (AoC) was computed by estimating the area under the fitted dose-response curve by the trapezoidal rule within ranges of relative growth rates compared to untreated controls between 0% and 100%, and within ranges of drug concentrations between 1 nM and 1 mM, and dividing the estimated area by the sum of areas below and above the curve. The relative AoC measure used in this work thus captures both the potency of a compound combination (usually measured by IC50 or GI50) as well as the maximum effect on cellular growth (as measured by the minimum of the curve); the relative AoC is of particular usefulness for capturing the efficacy of DDR inhibitors, many of which often have a comparatively low maximum effect less than 50% growth inhibition at realistic concentrations, which makes IC50 and GI50 less practically relevant.<br>Combination effects for the different compound combinations are calculated using the Bliss independence model 64,65 under the assumption of independent modes of action of the combination partners. Bliss excess was calculated as the average excess of the observed effect EOBS (i.e., the relative reduction of growth rate compared to untreated controls) over the calculated linear combination of the monotherapy treatments effects (E1+2 = E1 + E2 – E1 E2) for all concentrations used:<br>Eq. (1)<br>In this formulation, the Blissexcess is a continuous value between -1 and 1 where values higher than about 0.2 are usually considered synergistic, and values below about -0.2 are usually considered antagonistic.<br>Quantification and statistical analysis for drug response variance test<br>For hierarchical clustering based on drug responses, we used heatmap.2 function of gplots module (3.1.3) from R (4.2.3) for hierarchical clustering using euclidean as the distance function and ward.D2 as the cluster function.<br>We used Python (>=3.8) module scipy (1.11.3) to carry out the Kruskal-Wallis test to test if a drug has different responses between different cancer types. Kruskal-Wallis test is especially suitable for this situation as a non-parametric test, so it won't be affected by the different sample sizes of the subsets. For the significantly tissue-specific drugs (p<0.01), we also used scipy to carry out post-hoc tests, including Dunn's test, Mann-Whitney Pairwise test, Conover-Iman test and bootstrapping for 10,000 times to locate the significantly different tissue types. Bonferroni correction was performed to adjust the above multiple comparisons. The versions of all software that has been used above can be found in Table S6.

For manuscripts utilizing custom algorithms or software that are central to the research but not yet described in published literature, software must be made available to editors and reviewers. We strongly encourage code deposition in a community repository (e.g. GitHub). See the Nature Portfolio guidelines for submitting code & software for further information.

Data

Policy information about availability of data

All manuscripts must include a data availability statement. This statement should provide the following information, where applicable:

- Accession codes, unique identifiers, or web links for publicly available datasets
- A description of any restrictions on data availability
- For clinical datasets or third party data, please ensure that the statement adheres to our policy

The DDR combination in vitro screening data collected in this study are shared at and can be freely downloaded from: https://osf.io/8hbsx/. Source data are provided with this paper.

Research involving human participants, their data, or biological material

Policy information about studies with human participants or human data. See also policy information about sex, gender (identity/presentation), and sexual orientation and race, ethnicity and racism.

Reporting on sex and gender	Out study is conducted on purchased cancer cell lines and gender and sex related analysis is not relevant to our study.
Reporting on race, ethnicity, or other socially relevant groupings	See above.
Population characteristics	See above.
Recruitment	See above.
Ethics oversight	Merck Healthcare KGaA and the University of Michigan.

Note that full information on the approval of the study protocol must also be provided in the manuscript.

Field-specific reporting

Please select the one below that is the best fit for your research. If you are not sure, read the appropriate sections before making your selection.

☒ Life sciences ☐ Behavioural & social sciences ☐ Ecological, evolutionary & environmental sciences

For a reference copy of the document with all sections, see nature.com/documents/nr-reporting-summary-flat.pdf

Life sciences study design

All studies must disclose on these points even when the disclosure is negative.

Sample size	We carried out the largest DDR targeted screening so far including a total of a total of 17,912 combination treatment experiments.
Data exclusions	No data were excluded from the analysis.
Replication	Each drug combination-cell line experiment has 1-4 replicates. All experiments with more than one replicates were used to calculate the reproducibility of the dataset. For the AoC score for monotherapy, the Pearson's correlation = 0.8380 (p < 1e-22) between replicates, and for combination treatment, the bliss score shows Peason's correlation = 0.7611 (p < 1e-22) between replicates.
Randomization	Randomization is not relevant to our study. Our study is not clinical study and did not need to select experimental control.
Blinding	Blinding is not needed in this study since it's not clinical study and does not involve group allocation.

Reporting for specific materials, systems and methods

We require information from authors about some types of materials, experimental systems and methods used in many studies. Here, indicate whether each material, system or method listed is relevant to your study. If you are not sure if a list item applies to your research, read the appropriate section before selecting a response.

Materials & experimental systems

n/a	Involved in the study	
☒	☐	Antibodies
☐	☒	Eukaryotic cell lines
☒	☐	Palaeontology and archaeology
☒	☐	Animals and other organisms
☒	☐	Clinical data
☒	☐	Dual use research of concern
☒	☐	Plants

Methods

n/a	Involved in the study	
☒	☐	ChIP-seq
☒	☐	Flow cytometry
☒	☐	MRI-based neuroimaging

Eukaryotic cell lines

Policy information about cell lines and Sex and Gender in Research

Cell line source(s)	All 62 cell lines used in this study (A204, A2780, A549, ASPC1, BXPC3, CACO2, COLO205, DLD1, DU145, EFO21, EJ28, HCT15, HEPG2, HS578T, HS729, HT1080, IGROV1, J82, JIMT1, LOVO, MDAMB231, MDAMB435, MDAMB436, MDAMB468, MHHES1, MIAPACA2, MT3, NCIH292, NCIH358M, NCIH460, NCIH82, OVCAR3, OVCAR4, PANC1, PC3, PLCPRF5, RD, RDES, SAOS2, SF268, SF295, SKBR3, SKLMS1, SKMEL28, SKMEL5, SW620, T24, TE671, U2OS, U87MG, UMUC3, HL60, KASUMI1, L363, MINO, MV411, RAMOS, SUDHL10, SUDHL6, THP1, WSUNHL) were purchased directly from the ATCC, NCI, CLS and DSMZ cell line collections. The cell lines were grown in the media recommended by the suppliers in the presence of 100 U/ml penicillinG and 100 µg/ml streptomycin supplied with 10% FCS.
Authentication	The cell lines used in this study were authenticated by genome sequencing. The qualified genomic DNA of the cell line samples was fragmented by an ultrasonicator (Covaris). By adjusting shearing parameters, DNA fragments were concentrated in 500bp peaks for each sample. These fragments were purified, end blunted, 'A' tailed, and adaptor-ligated. DNA templates with adapters were then selectively enriched using PCR in order to obtain a sufficient amount for the DNA library. The concentration of the libraries was quantified by a bioanalyzer (Agilent Technologies) and real-time PCR method. Each qualified DNA library was sequenced on the Illumina HiSeq platform using paired-end reads according to Illumina manufacturer's instructions. Sequencing-derived raw image files were processed by Illumina base calling Software for base-calling with default parameters and the sequence data of each cell line was generated using an Illumina HiSeq 2000 instrument in paired-end mode at 2 × 100 bp read length. Subsequently, short nucleotide variations and copy number variations were computed using VarDict 98 and CNVkit 98,99, respectively, in the bcbio workflow system 100 using default parameters against the human reference genome hg19 with Ensembl 75 gene annotations. Variant calling by VarDict was conducted by requiring a minor allele frequency of at least 10% and minimal support for four de-deduplicated reads for each variant call; in addition, calibrated filters for strand bias, mean position of the variant in read, minimum mean base quality, NM/MQ mapping qualities, and DP/QUAL variant qualities were applied as per the bcbio default configuration.
Mycoplasma contamination	All cell lines were tested negative for mycoplasma contamination.
Commonly misidentified lines (See ICLAC register)	No commonly misidentified lines.

Plants

Seed stocks	*Report on the source of all seed stocks or other plant material used. If applicable, state the seed stock centre and catalogue number. If plant specimens were collected from the field, describe the collection location, date and sampling procedures.*
Novel plant genotypes	*Describe the methods by which all novel plant genotypes were produced. This includes those generated by transgenic approaches, gene editing, chemical/radiation-based mutagenesis and hybridization. For transgenic lines, describe the transformation method, the number of independent lines analyzed and the generation upon which experiments were performed. For gene-edited lines, describe the editor used, the endogenous sequence targeted for editing, the targeting guide RNA sequence (if applicable) and how the editor was applied.*
Authentication	*Describe any authentication procedures for each seed stock used or novel genotype generated. Describe any experiments used to assess the effect of a mutation and, where applicable, how potential secondary effects (e.g. second site T-DNA insertions, mosiacism, off-target gene editing) were examined.*
